# Development of Radiomic-Based Model to Predict Clinical Outcomes in Non-Small Cell Lung Cancer Patients Treated with Immunotherapy

**DOI:** 10.3390/cancers14235931

**Published:** 2022-11-30

**Authors:** Olena Tankyevych, Flora Trousset, Claire Latappy, Moran Berraho, Julien Dutilh, Jean Pierre Tasu, Corinne Lamour, Catherine Cheze Le Rest

**Affiliations:** 1 Nuclear Medicine Department, Poitiers University Hospital, 86000 Poitiers, France; 2 LaTIM, INSERM, UMR 1101, 29200 Brest, France; 3 Oncology Department, Poitiers University Hospital, 86000 Poitiers, France; 4 Medical School, University of Poitiers, 86000 Poitiers, France; 5 Radiology Department, Poitiers University Hospital, 86000 Poitiers, France

**Keywords:** PET/CT, radiomics, NSCLC, immunotherapy, response to therapy, durable clinical benefit, survival

## Abstract

**Simple Summary:**

In locally advanced or metastatic non-small cell lung cancer (NSCLC), immunotherapy has become a standard as it can improve overall survival and progression-free survival. However, a durable clinical benefit (DCB) is only achieved in 20–50% of patients. Early identification of patients likely to benefit from this treatment is not only challenging but also crucial to avoid immune-related toxicities in patients unlikely to achieve DCB. The aim of our retrospective study was to assess the value of baseline and serial FDG-PET/CT radiomics for the prediction of response and survival in NSCLC patients undergoing immunotherapy. In a group of 83 patients, multimodality radiomics and delta-radiomics models provided added predictive value compared to conventional clinical parameters. Multimodality radiomics-based models developed using appropriate machine learning processes were able to predict progression, DCB, Overall Survival and Progression Free Survival with high confidence.

**Abstract:**

Purpose: We aimed to assess the ability of radiomics features extracted from baseline (PET/CT0) and follow-up PET/CT scans, as well as their evolution (delta-radiomics), to predict clinical outcome (durable clinical benefit (DCB), progression, response to therapy, OS and PFS) in non-small cell lung cancer (NSCLC) patients treated with immunotherapy. Methods: 83 NSCLC patients treated with immunotherapy who underwent a baseline PET/CT were retrospectively included. Response was assessed at 6–8 weeks (PET/CT1) using PERCIST criteria and at 3 months with iPERCIST (PET/CT2) or RECIST 1.1 criteria using CT. The predictive performance of clinical parameters (CP), standard PET metrics (SUV, Metabolic Tumor volume, Total Lesion Glycolysis), delta-radiomics and PET and CT radiomics features extracted at baseline and during follow-up were studied. Seven multivariate models with different combinations of CP and radiomics were trained on a subset of patients (75%) using least absolute shrinkage, selection operator (LASSO) and random forest classification with 10-fold cross-validation to predict outcome. Model validation was performed on the remaining patients (25%). Overall and progression-free survival was also performed by Kaplan–Meier survival analysis. Results: Numerous radiomics and delta-radiomics parameters had a high individual predictive value of patient outcome with areas under receiver operating characteristics curves (AUCs) >0.80. Their performance was superior to that of CP and standard PET metrics. Several multivariate models were also promising, especially for the prediction of progression (AUCs of 1 and 0.96 for the training and testing subsets with the PET-CT model (PET/CT0)) or DCB (AUCs of 0.85 and 0.83 with the PET-CT-CP model (PET/CT0)). Conclusions: Delta-radiomics and radiomics features extracted from baseline and follow-up PET/CT images could predict outcome in NSCLC patients treated with immunotherapy and identify patients who would benefit from this new standard. These data reinforce the rationale for the use of advanced image analysis of PET/CT scans to further improve personalized treatment management in advanced NSCLC.

## 1. Introduction

Lung cancer is the leading cause of cancer-related death worldwide [1]. Non-small cell lung cancer (NSCLC) accounts for 80–90% of primary lung cancers, and is mostly diagnosed at an advanced stage with prognosis remaining poor despite recent therapeutic advances [2]. The introduction of immunotherapy for the treatment of locally advanced or metastatic non-small cell lung cancer (NSCLC) has shown an improvement in terms of overall survival and progression-free survival. However, a durable clinical benefit (DCB) (>6 months) is only achieved in 20–50% of patients [3]. Early identification of patients likely to benefit from this treatment is therefore crucial. In addition, newly described response patterns (pseudo-progression, hyper-progression) make response assessment even more challenging [4]. Therefore, robust response-predictive biomarkers at baseline are crucial to avoid immune-related toxicities in patients unlikely to achieve DCB. 

PD-L1 status has been an important element in treatment decision making until now. However, approximately 10% of patients whose tumors do not express PD-L1 respond to treatment, and response is also not certain in cases of high expression of this biomarker [3,5,6,7,8]. Alternative biomarker identification is an active research domain in which medical imaging plays an increasingly important role. 

18F-FDG PET/CT is commonly used for characterization and staging of lung cancers. Medical images are easy to repeat over time, non-invasive and promising for personalized patient management by reflecting tumor heterogeneity, which seems to be a major cause of the disparity between response and prognosis. However, for such applications, quantitative analysis is required.

Radiomics is an approach that allows a finer characterization of tumor lesions by extracting numerous quantitative parameters from medical images (CT, PET/CT and MRI, for example) [9]. These features can be used to build complex mathematical models for lesion characterization (benign versus malignant lesions [7,8,10,11,12], histology [13] and for the prediction of patient outcome [14,15]. In the majority of these studies, hand-crafted features extracted from the segmented functional or fused image tumor volumes have been considered. More recently, some studies have also considered the use of deep learning models in order to directly classify lung cancer patients in terms of overall survival without explicitly extracting radiomics features [16]. All these studies have shown some promising results, but in both cases, there are no specific investigations addressing the use of PET/CT radiomics for patients undergoing immunotherapy treatment. 

The changes in these features over time (delta-radiomics) have also been suggested to obtain a more accurate evaluation of tumor response [17]. While single time-point radiomics and delta-radiomics showed promising results in helping clinicians to choose the most appropriate treatment in various cancer models, there are still only limited data on the potential value of using these features in the evaluation of NSCLC patients undergoing immunotherapy using CT [18]. Mu et al. were recently the first to test a multiparametric radiomic model combining baseline PET and CT features to predict tumor response and survival [19]. Using advanced statistical analyses, they reported more heterogeneous tumors to have a greater probability of reaching DCB, but they did not investigate the potential additional value of serial examinations.

In this study we aim to assess the ability of radiomics features extracted from baseline and follow-up 18F-FDG PET/CT scans, as well as their changes during treatment, to predict response to immunotherapy, DCB, overall survival and progression-free survival. 

## 2. Materials and Methods

### 2.1. Patient Population

In this retrospective study, data were retrieved from a total of 83 patients treated at Poitiers University Hospital (France) between September 2016 and December 2020. Inclusion criteria were as follows: Eastern Cooperative Oncology Group (ECOG) performance status (PS) ≤ 2, histologically proven NSCLC treated with anti-PD-L1 immunotherapy (nivolumab, pembrolizumab or atezolizumab, as monotherapy or associated with chemotherapy) and available baseline 18F-FDG PET/CT (PET/CT0) performed within 3 months before the initiation of immunotherapy. 

Treatment was chosen depending on histology, PD-L1 expression, ECOG PS and risk factors and approved by the multidisciplinary oncological board. Immunotherapy was introduced as a first-line therapy in 23 patients (28%). Ten patients had PD-L1 expression higher than 50% and received pembrolizumab alone (*n* = 8) or combined with chemotherapy (*n* = 2). Thirteen patients were treated with pembrolizumab associated with platinum-based chemotherapy (cisplatin or carboplatin) and pemetrexed. Two patients received atezolizumab, either alone (*n* = 1) or associated with carboplatin and etoposide (PD-L1 expression not performed). Sixty patients (72%) received immunotherapy for recurrence or failure after at least one line of chemotherapy. Seventeen of them had PD-L1 expression ≥ 1% and received pembrolizumab, 41 received nivolumab and 3 received atezolizumab. Five patients were treated with a combination of pembrolizumab, carboplatin and pemetrexed. Immunotherapy administration followed the recommendations for each molecule at the time of treatment.

Baseline clinical, demographic and biological data were retrieved from medical records (age, sex, performance status, smoking history, histological subtype, stage (8th TNM classification of the International Association for the Study of Lung Cancer [20]), presence of brain metastasis or not, previous treatments and PD-L1 status (if available).

All patients gave their informed consent for the use of their personal and clinical data. No ethical committee approval was required, given the retrospective nature of this study of previously anonymized data.

### 2.2. Image Acquisition, Segmentation, Pre-Processing and Feature Extraction

Eighteen F-FDG PET/CT scans were performed following EANM guidelines. Acquisition parameters are described in Supplementary Materials. The largest lung lesion was semi-automatically segmented using the Fuzzy Locally Adaptive Bayesian (FLAB) algorithm [20], and the obtained volume of interest was adjusted manually by an experienced clinician, if needed. The performance of this algorithm has been extensively evaluated for functional tumor segmentation, demonstrating high reproducibility and robustness for different cancer models [21,22,23]. Tumor segmentation on CT images was performed by applying the PET VOI on CT images using 3D Slicer [24].

Prior to feature extraction, pre-processing operations were applied on PET and CT images, as described in Supplementary Materials, to take into account the variability of image acquisition (PET/CT scanners, acquisition protocols). 

Metabolic and conventional volumetric parameters were extracted from CT and metabolic VOIs (including SUVmax, SUVmin, SUVmean, Metabolic Tumor Volume (MTV) and Total Lesion Glycolysis (TLG)). In addition, a total of 2430 radiomic features were extracted from both CT and PET VOIs using Pyradiomics [25], 107 were derived from original images and 2323 were derived from preprocessed images (using different combinations of interpolation, resampling and filtering), including: 2D and 3D shape parameters, first-order (histogram-based characteristics) and second-order (texture parameters) features. Detailed parameter definitions are available at the Pyradiomics website, and the list is presented in Table S1 [25].

### 2.3. Patient Outcomes

#### 2.3.1. Follow-Up and Response Assessment

Patient follow-up was based on clinical examination every 12 weeks until disease progression or death occurred. Metabolic response was assessed using PERCIST criteria on follow-up PET/CT performed 6 to 8 weeks (PET/CT1) after treatment initiation [26]. Response was defined as: complete metabolic response (CMR), partial metabolic response (PMR), stable metabolic disease (SMD) or progressive metabolic disease (PMD), considering the most hypermetabolic lesion on each scan. 

In cases of new lesions on PET/CT1 without clinical worsening, the response was qualified as unconfirmed progression (UPMD). Then, discrimination between true progressive disease and pseudo-progression was carried out using a second follow-up PET/CT (PET/CT2) performed after an additional month of treatment [27].

Response was also determined on follow-up CT performed at 8 to 12 weeks according to Response Evaluation Criteria in Solid Tumors version 1.1 (RECIST) [28]. When patients had no follow-up PET/CT, response was only assessed using RECIST. Patients were classified as responders if response was defined as either complete response (CR), partial response (PR) or stable disease (SD). Patients with progressive disease were considered as non-responders. Patients were treated until clinical worsening, confirmed progression or unacceptable toxicity.

#### 2.3.2. Survival and Durable Clinical Benefit (DCB)

Overall survival (OS) was calculated as the time from initiation of immunotherapy to death, censored at the date of last follow-up for survivors. Progression-free survival (PFS) was determined as the time from treatment initiation to disease progression or death, censored at the date of last follow-up for survivors without progression. Durable clinical benefit (DCB) to immunotherapy was defined as alive and without disease progression at 6 months.

### 2.4. Statistical Analysis

We evaluated the discriminative power of clinical parameters (age, sex, smoking, ECOG PS, histology, PDL1, stage, previous treatment, immunotherapy line and molecule, tumor response) to predict the studied endpoints (OS, PFS, response, DCB) using chi-square and Mann–Whitney tests for quantitative and qualitative variables, respectively, with *p*-values < 0.05 considered to be statistically significant.

The Kaplan–Meier analyses with log-rank tests were performed for PFS and OS (Tables S5–S9).

To avoid overfitting, we discarded highly correlated features using Spearman’s rank correlation, followed by a feature selection using Least Absolute Shrinkage and Selection Operator (LASSO) [29]. Area under the receiver operating characteristic curve (AUC) analysis, estimated using univariate logistic regression with Bootstrap, was then performed to evaluate the predictive value of each parameter (clinical parameters (CP), standard PET metrics and radiomics features from baseline and follow-up PET/CT) for the different endpoints.

In the multivariate analysis, 7 models (CP, PET, CT, PET-CP, CT-CP, PET-CT-CP) were built with the most promising selected features using the Random Forest classification method, with 10-fold cross validation repeated 10 times on the training set (75% of the initial data) for hyperparameters tuning with randomized patients split (100 iterations for sample bias correction). The number of variables selected was part of the model tuning process. Number of features varied between 3 and 8, with an increment of 1 for each. So, for each model, this number was unique, selected upon the best training results. Subsequently, the selected model (set of hyperparameters and selected features) was applied on the test sample (25% of the initial data). The performance of each model in predicting outcome was measured by AUC analysis.

The delta-radiomics features were defined as the relative net change between two images: relative Net Change = (FeatureT1−FeatureT0)/FeatureT0. Here, FeatureT0 was the value of a feature before the treatment and FeatureT1 was the value of the feature at the 2-month evaluation of the treatment. The delta-radiomics predictive power was also tested using the same methodology. All the analyses were performed with standard Python modules, including Sklearn, pandas and Matplotlib.

## 3. Results

### 3.1. Patients Characteristics

Clinical and demographic characteristics of the 83 patients enrolled in this study are summarized in Table 1. No patient was lost at follow-up.

Clinical parameters were not significantly different in responders and non-responders. Considering demographic data, ECOG performance status (PS) was associated with significant differences in OS and PFS. PFS and OS were significantly shorter in patients with PS ≥ 1 (*p* = 0.0005 and *p* = 0.003, respectively). PS and stage before immunotherapy were also significantly lower in patients who obtained DCB (*p* = 0.00009 and *p* = 0.03, respectively) (Table S3).

Survival tended to be longer in patients more than 63 years old (Figure S1A). 

### 3.2. Patient Outcomes

The median follow-up was 865 days (931 ± 569, range 98–1759 days). At the last follow-up, 40 patients (48%) were still alive and 18 (22%) were still receiving immunotherapy. Nine patients (11%) experienced immunotherapy-induced adverse effects (five with thyroiditis, one with rising liver enzymes, one with pneumonitis, one with Raynaud’s syndrome and one with sensitive neuropathy). 

Immunotherapy was stopped in 65 patients for various reasons: progression or clinical worsening in 49 patients (75%); unacceptable adverse event in four patients (6%); introduction of a high dose of corticosteroids (because of brain metastasis with edema) in four patients (6%); second cancer requiring the introduction of another chemotherapy in one patient (2%); 2 years of clinical benefit with immunotherapy in seven patients (11%).

Metabolic response was studied using PET/CT1 in 71 patients (85%), and 34 of them underwent a second PET/CT one month later (PET/CT2). According to iPERCIST criteria, metabolic response after 3 months of treatment was as follows: CMR in three patients (4%), PMR in 29 patients (41%), SMD in eight patients (11%) and PMD in 31 patients (44%). Responder rate was 56% (40 patients). Out of the 40 responders, four were first assessed as PMD based on PET/CT1 and reclassified as either PMR (*n* = 3) or SMD (*n* = 1) using PET/CT2. In these cases of pseudo-progression, the patients ultimately obtained a durable clinical benefit (DCB). Response was evaluated using only RECIST 1.1 in 12 patients (PET/CT1 was not performed) and was as follows: 5 PR, 4 SD and 3 PD. A total of 49 patients were considered as responders (59%) and 34 as non-responders (41%) after three months of therapy. DCB was then obtained in 35 patients (42%).

Outcome was more favorable for responders than non-responders, with a longer median PFS (250 days vs. 52, *p* <0.001) and OS (351 days vs. 194 *p* <0.001) (Table 1).

### 3.3. Radiomics and Prediction of Progression, DCB and Survival

#### 3.3.1. Univariate Analysis

Best results of radiomics features extracted from the largest lung lesion at baseline (PET/CT0) and 2 months (PET/CT1) after treatment initiation are presented in Tables S2–S4 and Figures S1 and S2. Predictive value of parameters derived from follow-up PET/CT scns performed at month 3 will not be described in this paper as the sample size (34 patients) was too small to obtain reliable results. The performance of radiomics parameters in predicting response at month 2 (PET/CT using PERCIST) and month 3 (using iPERCIST for PET/CT and RECIST for CT) were studied but will not be detailed further in the following sections, given that they were similar to those predicting other primary endpoints. Therefore, only progression, DCB and survival results are considered in the results below.

##### At Baseline (PET/CT0)

Standard PET-based features (SUVs, MTV, TLG) were not able to significantly discriminate between patients in terms of progression, DCB or survival (*p* = 0.44). Predictive performance of clinical parameters and standard PET/CT metrics were lower. The predictive power of ECOG PS and age were moderate with AUC of 0.70 for DCB and OS. AUC for volume and TLG did not exceed 0.67 to predict progression. SUV and all the other clinical features had low predictive power.

Advanced image analysis provided better results. For example, longer PFS and OS were observed in cases of high PET0_GLCM_Clustershade (*p* = 0.0007), low CT0_kurtosis (*p* = 0.001) and low CT0_GLSZM_GLNUN (*p* = 0.00003). 

Moreover, PET- and CT-based radiomics features were able to predict outcomes with AUC up to 0.85 for progression and 0.80 for OS. Texture-based features had better performance than first-order and shape parameters. The highest predictive power was obtained with texture-based parameters representing regional or local heterogeneity characterization (such as CT0_GLRLM_LRLGLE (AUC 0.85) and PET0_GLCM_Imc1 (AUC 0.85) for progression, CT0_GLDM_SDE (AUC 0.80) and CT0_GLSZM_ ZonePercentage (AUC 0.80) for OS). Radiomics predictive power was moderate for PFS and DCB (AUC up to 0.74 for the best parameters).

##### At Month 2 (PET/CT1)

OS and PFS were significantly longer in patients with more homogeneous tumors, as demonstrated by low GLCM-Imc1 (*p* = 0.00003, HR = 5.99, for TEP1), low kurtosis (*p* = 0.00007, HR = 3.79 for CT1), low busyness (*p* = 0.0008, HR = 2.94 for CT1), high GLCM-correlation (*p* = 0.0002, HR = 0.30 for CT1) and high NGTDM-coarseness (*p* = 0.004, HR = 0.37 for CT1). OS and PFS were also significantly longer in patients with more regular tumors (high Surface-Volume Ratio, *p* = 0.0004 for CT1; high sphericity, *p* = 0.000006, for PET1).

Performance of clinical parameters and standard PET/CT metrics to predict outcome remained low, similarly to those at baseline. A higher number of radiomics features demonstrated a high predictive value (as demonstrated by an AUC ≥ 0.80), especially for progression, with AUC up to 0.91 (Variance for CT1) and 0.88 (PET1_Maximum), for example. After 2 months of treatment and unlike at baseline, first-order parameters (extracted from filtered images) managed to perform equally to shape-based and some more complex texture-based features. Radiomics overall predictive performances at month 2 were higher than at baseline, except for the prediction of DCB.

##### Delta-Radiomics

Baseline and follow-up PET/CT studies were used to determine radiomics features variation during treatment (delta-radiomics). None of these features achieved higher predictive performance than parameters at month 2 (PET/CT1). Increasing size of the lesion (as reflected by the least axis length (*p* = 0.00006) and mesh volume (*p* = 0.0008)) was associated with non-response. We also observed that non-responder tumors tended to lose the roundness of their shape, with a decrease in sphericity (*p* = 0.1) and flatness (*p* = 0.16), but these changes were not statistically significant.

DeltaPET parameters demonstrated an equally high predictive power for PFS and OS (AUC = 0.81 for deltaPET_GLDM_LGLE and AUC = 0.83 for deltaPET GLRLM_RunEntropy, respectively) compared with PET/CT1 features (AUC = 0.83 with PET1_GLCM_ClusterTendency, AUC = 0.84 with CT1_GLCM_Imc2 for PFS, and AUC = 0.82 for PET1_GLRLM_SRLGLE for OS). DeltaCT features had a slightly lower predictive power for OS compared with PET/CT1 features (AUC = 0.73 for deltaCT_GLRLM_LRLGLE and AUC = 0.81 for CT1_GLSZM_SZNU). The performance of delta-radiomics features were better than baseline parameters in predicting PFS (AUC of 0.84 for deltaCT, 0.81 for deltaPET and 0.74 and 0.73 for TEP0 and CT0, respectively). They did not perform better than single time-point features for DCB (AUC of 0.77 and 0.73 for deltaPET and deltaCT and AUC of 0.74 and 0.75 for PET0 and CT0, respectively) or for progression (AUC = 0.82 for deltaCT_flatness and 0.74 for deltaPET_GLCM_Correlation).

#### 3.3.2. Multivariate Analysis

**Figure 1:**
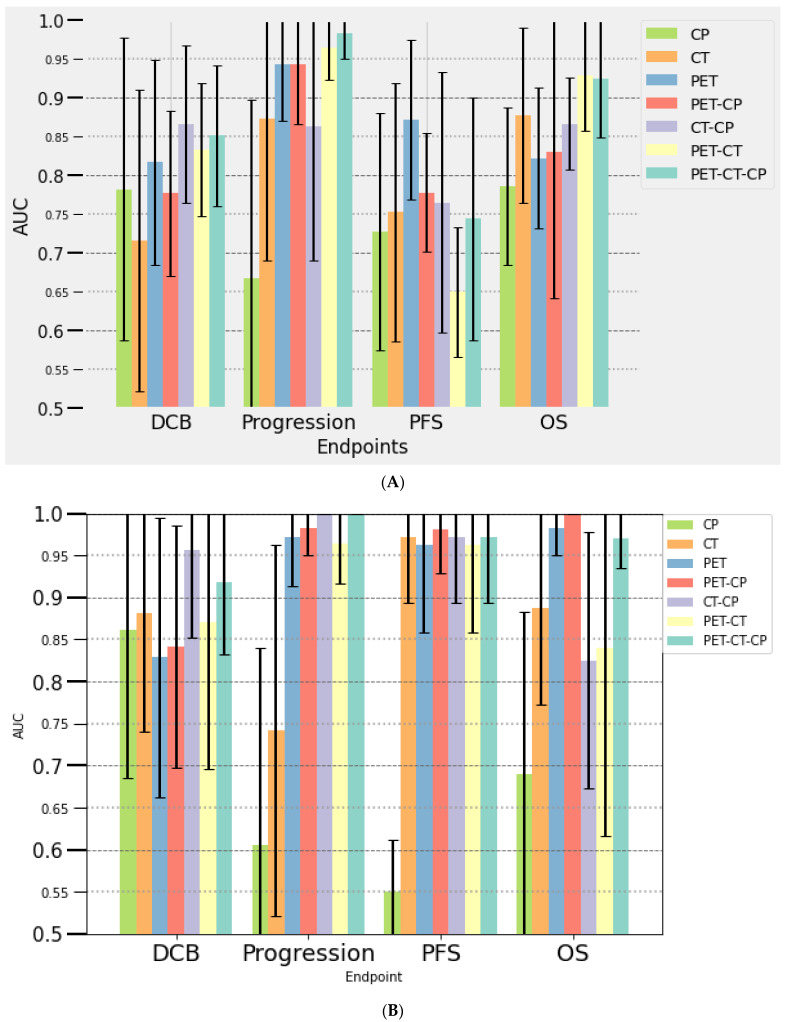
Performances of seven multi-parametric models for the prediction of outcome using clinical data (CP) and radiomics features derived from PET and CT at baseline (**A**) and at month 2 (**B**) in the training data set, with their standard deviations integrated. OS: overall survival; PFS: progression-free survival, DCB: durable clinical benefit.

**Figure 2:**
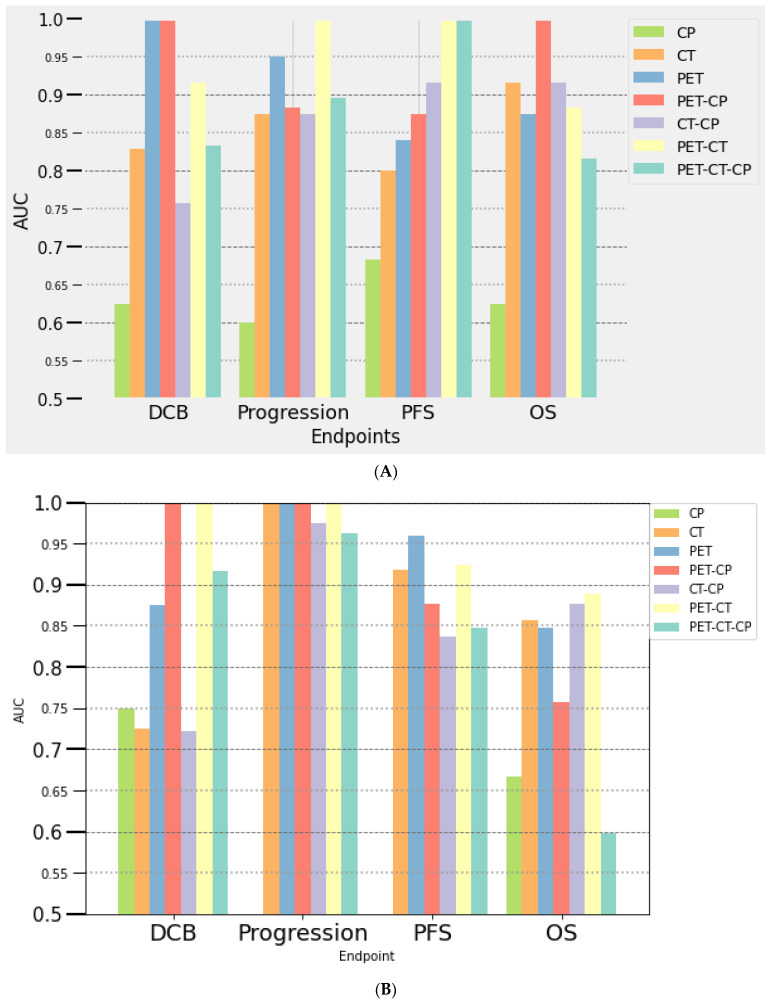
Performances of seven multi-parametric models for the prediction of outcome using clinical data (CP) and radiomics features derived from PET and CT at baseline (**A**) and at month 2 (**B**) in the testing dataset.

Seven multivariate prediction models were composed of a variable number of selected features (from 3 to 18): clinical parameters alone (CP), PET- and CT-derived parameters alone or combined with CP. The results are presented in Figure 1 (training) and Figure 2 (testing). The radiomics-based models consisted only of single-time point features (TEP/CT0 or TEP/CT1), given that delta-radiomics did not show higher performances. The metabolic response determined using PERCIST on PET/CT1 was integrated into the models established at month 2. The performances in predicting response to immunotherapy (assessed with PERCIST, iPERCIST and RECIST) of each model were also investigated and were similar to those of other outcomes (DCB, progression, survival) and therefore will not be further detailed. 

##### At Baseline (PET/CT0)

Clinical parameters model showed low predictive value for progression, DCB, PFS and OS (AUC of 0.64, 0.40, 0.81, 0.5, and 0.67, 0.60, 0.71, 0.63 in the training and the testing subset, respectively) (Figure 1A and Figure 2A). Among available clinical parameters, only ECOG PS was selected for DCB and PFS and only age for OS in the process of model building. Models including radiomics features had a high predictive value outcome with AUCs over 0.80, except for the CT-CP model (AUC of 0.75 for the prediction of DCB in the testing subset). The PET radiomics model outperformed the CT model in predicting DCB, progression and PFS. Combining PET and CT features further improved performance for the prediction of DCB, progression and OS, compared to PET or CT features-only models. The most contributing radiomic features to the models were first-order parameters extracted from filtered CT images and texture features extracted from PET and CT images (such as skewness, median, NGTDM_Complexity, GLCM_Autocorrelation and GLCM_imc1). During the building process, AUC varied greatly for some models, with standard deviation up to 15% for the PET-CT-CP model predicting PFS (Figure 1). Highest stability (SD = 3%) was obtained predicting progression using PET-CT (AUC of 0.96 and 1 for the training and validation subsets, respectively), DCB using PET (AUC of 0.82 and 1) and PET-CP (AUC of 0.78 and 1), PFS using PET-CT (AUC of 0.65 and 1) and PET-CT-CP (AUC of 0.74 and 1) and OS using PET-CP (AUC of 0.83 and 1). 

##### At Month 2 (PET/CT1)

We observed a similar trend compared with the results obtained at baseline (Figure 1B and Figure 2B). The CP model was less efficient than models including radiomics features. There was an overall increase of prediction performances for most of the multivariate models for progression, PFS and OS, in addition to more consistent results between the training and testing subsets, especially for progression and PFS. The models that appeared to be the most promising for the different outcomes were: PET-PC (AUC of 0.91 and 1 for the training and testing subsets, respectively) and PET-CT (AUC of 0.84 and 1) for DCB; PET (AUC of 0.97 and 1), CT (AUC of 0.74 and 1), PET-PC (AUC of 1 and 1) and PET-CT (AUC of 0.96 and 1) for progression; PET for PFS (AUC of 0.96 and 0.96), and PET-CT for OS (AUC of 0.84 and 0.89).

The most contributing features to the models were textural features, first-order parameters extracted from filtered images (PET and CT), age, ECOG PS and response assessed with PERCIST on PET/CT.

We have performed an analysis of the radiomics quality, according to which our study scores19 points (out of 36, Table S10) [30].

## 4. Discussion

The action of anti-PD-1 and anti-PD-L1 antibodies differs from conventional chemotherapies and has induced new evolution patterns, making imaging-based response assessment more challenging. Response may be delayed, and early tumor infiltration by immune cells may induce an initial increase in lesion size. In order to take into account these atypical patterns, we chose to evaluate patients according to iPERCIST criteria, as proposed by Goldfarb et al. in 2019 [27]. We identified four patients (5%) with pseudo-progressions, which is consistent with previous studies in relation to a temporary increase in tumor burden to transient immune-cell infiltrate followed by tumor regression [31]. All of these patients subsequently obtained a lasting clinical benefit. This is again in agreement with previous studies reporting the occurrence of pseudo-progression being a good prognostic factor [32,33,34]. It is therefore important to be able to identify these response patterns to avoid stopping a potentially effective treatment too early.

We investigated the ability of clinical parameters and standard baseline PET/CT metrics (SUV, MTV, TLG) to discriminate between patients in terms of response (according to PERCIST at the first restaging, iPERCIST and RECIST1.1 after 3 months of treatment), DCB and survival. Low ECOG PS (PS = 0) and tumor stage (stage III vs. IV) were associated with DCB. These results are consistent with those of Seban et al. [35] and Nardone et al. [36]. In addition, OS was longer in patients with smoking history, in agreement with immunotherapy having been found to have a greater benefit in NSCLC patients with a smoking history than in those who had never smoked [37]. None of the considered clinical parameters was able to discriminate responders from non-responders.

Some authors previously reported a relationship between large MTV, high TLG and progression during immunotherapy, suggesting a potential predictive value of these parameters to predict response and survival [38,39]. In our study, these easily calculated features were not statistically different according to response or outcome, and therefore they did not reach a high level of predictive power, probably because of a narrower distribution of their values in our selected population. 

On the contrary, a large number of PET and CT radiomics features were correlated with therapeutic response, DCB and survival and had a high predictive value (AUC ≥ 0.80). The best-performing parameters at baseline were texture parameters. First-order and shape features also demonstrated a high predictive value, especially at the first restaging (PET1). 

Overall, PET and CT parameters extracted from PET/CT1 were greater predictors than those at baseline (with a gain ranging from 5 to 10% for the AUC of the best parameters for progression and PFS). Moreover, features at 4 months (PET2) showed AUCs up to 1 for the prediction of DCB and OS. This gain in predictive power should be taken with caution as there were fewer patients in the group studied at month 2 (71 segmented tumors) and month 3 (*n* = 34) compared with baseline, which could result in a higher risk of overfitting. 

OS and PFS were significantly longer in patients with more homogeneous tumors at baseline and at the first restaging. These results are in agreement with previous studies [36,38,40]. Nardone et al. [36] reported that high entropy related to tumor aggressiveness in the literature [41] and low correlation were both associated with poor prognosis. Ahn et al. also found that contrast and busyness were able to predict recurrence in surgically treated patients [42]. A low value of coarseness was an independent prognostic biomarker associated with a high risk of recurrence in our study. This finding is in contrast with Mu et al. [19] reporting patients with more heterogeneous tumors to have a higher probability of obtaining DCB.

In terms of shape, Mu et al. reported that patients with more convex tumors had a greater chance to achieve DCB [19]. Indeed, sphericity is thought to reflect PD-L1 expression and thus potential response to treatment, as PD-L1 cells could form rounder lesions according to Saeed-vafa et al. [43]. In our study, tumor shape also appeared to be significant for outcome, with longer PFS and OS in patients with more rounded but spiculated lesions on the first follow-up PET/CT, reflected by high sphericity and high Surface Volume Ratio, respectively. An increasing size of the lesion during the first 2 months was associated with non-response, as expected. Regarding tumor boundaries, Dercle et al. demonstrated that early regularization of tumor margins was strongly associated with response to nivolumab and gefitinib [44]. Complex tumor boundaries are indeed conventionally associated with tumor aggressiveness and worse outcome [45,46]. In our study, while non-responder tumors tended to lose the roundness of their shape, an increase in the smoothness of tumor contours (decrease in surface-to-volume ratio) was surprisingly associated with non-response. This atypical finding could result from the sensitivity of shape features to segmentation and respiratory motion. Deep learning used for feature extraction without the need for an intermediate segmentation step may be useful in the future to overcome such limitations [47].

Using PET-based multivariate modeling was more efficient in predicting DCB, progression and PFS, while CT remained a better predictor of OS. Those results underlined the complementarity of both imaging techniques. This was confirmed by an improvement of combined PET/CT models in predicting DCB, progression and OS. Unsurprisingly, the addition of low-performing clinical parameters did not further improvethe outcome prediction. It has been shown in prior studies that combined multi-modality models could perform better than single-modality approaches (clinical, radiomics or genomic data) for outcome prediction [48]. Overall, all multimodality models including radiomics features in the testing subset achieved a high predictive power (AUC ≥ 0.80) for all outcomes. The most significant radiomics features included in the models were texture features extracted from PET and CT images and first-order parameters extracted from filtered CT images. The results of the models whose performance varied the most between the training and testing subsets should be taken with caution. The models that appeared the most promising were therefore PET-CT-CP for DCB, PET-CT for progression and PET for PFS. The lowest variability of performance estimates (AUCs) was obtained for the prediction of progression, which is consistent with the highest predictive performance during the univariate analysis. The performances of these models are similar to those obtained by Mu et al. [19] in developing a radiomic signature to predict DCB in NSCLC patients treated with immunotherapy. They combined radiomics features extracted from PET, CT images and the fused PET and CT images (KLD), ECOG PS and histology with an AUC of 0.89 for the training dataset and AUCs of 0.86 in both the retrospective and prospective tests cohorts.

Our study had some limitations, including its retrospective design and the small sample size. The interval of time between baseline PET/CT and the introduction of immunotherapy in patients treated in our institution was variable, and we chose to only include patients with a maximum delay of 3 months (from a few days to 3 months maximum) to take into account the natural history of lung cancer and avoid non-representative baseline PET/CT results in a rapidly evolving pathology. This meaningful choice reduced our inclusion potential. However, in this study we applied a highly appropriate methodology to overcome the risk of overfitting, considering the large number of features extracted and the small size of the population. Indeed, to address this issue, we selected features using two steps. First, over-correlated parameters using Spearman rank coefficients were discarded. Then, the LASSO algorithm was used to select the most promising parameters as it is known to be a robust statistical method for data reduction [49]. In addition, we performed a cross-validation to build our models, which has been shown to be an efficient method to provide an unbiased estimation on survival prediction [50]. This rigorous methodology allowed us to consider a very large number of parameters to start with, and to avoid missing potentially important ones. Radiomics features extraction and analysis is complex and involves many steps that can affect the reproducibility of studies based on this approach [51]. After acquisition, segmentation is the first critical step for which we used a valid and robust method (FLAB) on PET images. Using a low-dose CT performed for PET attenuation correction, tumor segmentation on CT images was facilitated by automated alignment of the datasets from the acquisition system (PET/CT). Various filtering and quantization were applied to original datasets since no pre-processing consensus has yet been achieved, despite harmonization efforts from the scientific community. We partly addressed the issue of reproducibility and validation following image biomarker standardization and consensus–based definitions and guidelines for generating radiomics [52].

In this study, the median predicted value was used as a cut-off point to dichotomize high- and low-risk patients. It is likely that different values would yield different results. However, testing multiple cutoffs to find the best one without an independent validation dataset in which to test it has been repeatedly shown to yield overly optimistic results [49,50]. By using the median, this source of bias is avoided, and the conclusions remain conservative.

The fact that patient recruitment was carried out over several years (2016 to 2020) explained the heterogeneity of our population reflecting the gradual appearance of the various immunotherapy molecules and their combination with chemotherapy. Rapidly evolving practice resulted in immunotherapy being introduced in our population either in the first or second line with different treatment regimens. This resulting population heterogeneity is representative of that observed in clinical routine. The predictive value of PD-L1 status could not be investigated since it was not available for a third of our population, given that it was not necessarily requested for immunotherapy prescription, especially before 2019. 

Only 34 patients had a second follow-up PET/CT scan, given that this exam is not systematically recommended in routine for the follow-up of lung cancers. As demonstrated, it was found useful to better characterize initial progression, but it may be interesting to also suggest its use in a larger study based on our promising results for the prediction of DCB and OS.

Validation is always a crucial point in radiomics studies. To validate our predictive models, we divided our population into training and validation datasets. This choice necessarily led to a small-size validation group in which individual variability could explain slightly lower performance. 

## 5. Conclusions

The introduction of immunotherapy in the management of locally advanced and metastatic lung cancer has improved patient survival but only achieves a lasting clinical benefit in 20 to 50% of patients. The search for biomarkers that can successfully identify patients likely to benefit from this treatment is therefore a major challenge. Our study showed that radiomics parameters extracted from baseline and follow-up PET/CT scans, as well as their evolution during treatment, could play an important role in evaluating the risk of progression during treatment and in predicting response to therapy, DCB and survival of NSCLC patients treated with immunotherapy. As demonstrated in this real-clinical-conditions cohort, these parameters seemed to provide a real added value for outcome prediction compared with clinical or standard PET/CT metrics alone, which could be beneficial for personalized patient management. Although promising, these results provide the rationale for an external validation in a large prospective cohort to ensure their clinical significance.

## Figures and Tables

**Table 1:** Clinical and demographic characteristics of overall population (83 patients) and correlation of these parameters with iPERCIST response (evaluated in 71 patients) (chi-square and Mann–Whitney tests).

Characteristic	Overall Population (*n* = 83)	Responders (*n* = 40)	Non-Responders (*n* = 31)	*p* Value
**Age** (years), median (range)	63.5 (38–85)	61.5 (47–82)	65 (43–85)	0.28
**Sex**, *n* (%)				0.78
Men	59 (71)	31 (77)	22 (71)	
Women	24 (29)	9 (23)	9 (29)	
**Smoking**, *n* (%)				0.09
Smoker	79 (95)	40 (100)	29 (94)	
Non-smoker	4 (5)	0	2 (6)	
**ECOG PS**, *n* (%)				0.3
0	35 (42)	19 (47,5)	12 (39)	
1	44 (53)	19 (47,5)	18 (58)	
≥2	4 (5)	2 (5)	1 (3)	
**Histology**, *n* (%)				0.13
Adenocarcinoma	48 (58)	26 (65)	15	
Squamous cell carcinoma	22 (26)	8 (20)	11	
Other type	13 (16)	6 (15)	5	
**PD-L1**, *n* (%)				-
<1%	20 (24)	12 (30)	9 (29)	
1–49%	18 (22)	8 (20)	2 (6)	
≥50%	15 (18)	11 (28)	3 (10)	
Not performed	30 (36)	9 (22)	17 (55)	
**Stage before immunotherapy**, *n* (%)				0.27
IIB	1 (1)	1 (2)	0	
III	11 (13)	6 (15)	3 (10)	
IV	71 (86)	33 (83)	28 (90)	
**Previous treatment**, *n* (%)				
Surgery	19 (23)	11 (28)	7 (23)	
Radiotherapy	14 (17)	10 (25)	10 (32)	
Chemotherapy	58 (70)	22 (55)	26 (84)	
**Immunotherapy**, *n* (%)				
First line	23 (28)	24 (60)	26 (84)	
Second or >line	60 (72)	16 (40)	5 (16)	
Nivolumab	41 (49)	13 (33)	24 (78)	
Pembrolizumab	37 (45)	26 (65)	6 (19)	
Atezolizumab	5 (6)	1 (2)	1 (3)	
**PFS**, median (days)	156	250	52	<0.001
**DCB** , *n* (%)	35 (42)	25 (63)	3 (10)	<0.001
**OS**, median (days)	256	351	194	<0.001

ECOG PS: Eastern Cooperative Oncology Group performance status; OS: overall survival; PFS: progression-free survival.

## Data Availability

The data presented in this study are available on request from the corresponding author.

## Supplementary Materials

The following supporting information can be downloaded at: https://www.mdpi.com/article/10.3390/cancers14235931/s1. Table S1: Acquisition, reconstruction et preprocessing parameters; Table S2: List of radiomics parameters; Table S3: Results of descriptive analysis for OS, PFS and DCB; Table S4: Results of univariate analysis with bootstrap for durable clinical benefit (DCB) (mean AUC with standard deviation and confidence intervals); Table S5: Kaplan–Meier and Cox analysis scores for clinical parameters: (A) Progression-Free Survival, (B) Overall survival; Table S6: Progression-Free Survival Kaplan–Meier and Cox analysis scores for PET radiomics; Table S7: Overall Survival Kaplan–Meier and Cox analysis scores for PET radiomics; Table S8: Progression-Free Survival Kaplan–Meier and Cox analysis scores for CT; Table S9: Overall Survival Kaplan–Meier and Cox analysis scores for CT radiomics; Table S10: Radiomics Quality Score; Figure S1: OS and PFS (Kaplan–Meier and log rank test) related to (A) age, (B) performance status, (C) histology, (D) smoking history, (E) baseline metabolic tumor volume, (F) baseline total lesion glycolysis; Figure S2: Prediction of outcome: Best clinical, PET and CT parameters performance at (A) baseline and (B,C) during treatment: best AUC value obtained for the considered parameters using univariate analysis with bootstrap.
