# Supplementary material for: Development of Radiomic-Based Model to Predict Clinical Outcomes in Non-Small Cell Lung Cancer Patients Treated with Immunotherapy
Source: Cancers (Basel). 2022 Nov 30;14(23):5931. doi: 10.3390/cancers14235931 (PMC9739232; doi:10.3390/cancers14235931)
Supplement: Supplementary file 1 [file cancers-14-05931-s001.zip › cancers-1876276-supplementary.pdf]

**Table S1.** Acquisition reconstruction and pre processing parameters.

| | |
|---|---|
| **Acquisition parameter** | **Scans performed 60 ± 5 minutes after intravenous administration of 2.25 to 3 MBq/kg of 18F-FDG.**<br>**Scanner : Biograph mCT40 ToF (Siemens Healthcare, Erlangen, Germany).**<br>**Standard routine protocol based on EANM guidelines : fasting for at least 6 hours, glucose level <2 g/L.**<br>**Low-dose CT acquired for attenuation correction and anatomical correlation of PET abnormalities: tube current 120 kV, CARE Dose 4D current modulation system, reconstruction using 5 mm slice thickness.**<br>**PET acquisitions : 3.5 minutes per bed position from mid thighs to skull base.** |
| **Reconstruction** | OSEM-TrueX-TOF algorithm<br>3 iterations, 21 subsets<br>Voxel size : 4.073 x 4.073 x 2.072 $mm^3$ |
| **Pre processing** | Voxel size was first resampled to isotropic resolutions of 1 mm, 1.5 mm and 2 mm using cubic B-spline interpolation.<br>Various quantization methods were applied : fixed-bin width (0.25 and 0.5), fixed-bin number (32 and 64) of gray levels.<br>Filtering: Laplacian of Gaussian filters using four thresholds (0.5 mm, 1 mm, 2 mm, and 3 mm)<br>In addition, 8 different image series were also derived using Haar wavelet filters. |

**Table S2.** List of Radiomics Parameters.

| Parameter class | parameters |
|---|---|
| **First Order Features** | Energy<br>Total Energy .<br>Minimum<br>10th percentile<br>10th percentile<br>Maximum<br>Mean<br>Median<br>Interquartile Range<br>Range<br>Mean Absolute Deviation (MAD)<br>Robust Mean Absolute Deviation (rMAD) Root Mean Squared (RMS) Standard Deviation<br>Skewness<br>Kurtosis<br>Variance<br>Uniformity |
| **Shape Features (3D)** | Mesh<br>Surface<br>Surface Area to Volume ratio<br>Sphericity<br>Compactness<br>Spherical Disproportion<br>Maximum 3D diameter<br>Maximum 2D diameter (Slice)<br>Maximum 2D diameter (Column)<br>Maximum 2D diameter (Row)<br>Major Axis Length<br>Minor Axis Length<br>Least Axis Length<br>Elongation<br>Flatness |
| **Shape Features (2D)** | Mesh Surface |

| | |
|---|---|
| **Gray Level Co-occurrence Matrix (GLCM) Features**<br>**Local heterogenity** | Pixel Surface<br>Perimeter<br>Perimeter to Surface ratio<br>Sphericity<br>Spherical Disproportion<br>Maximum 2D diameter<br>Major Axis Length<br>Minor Axis Length<br>Elongation<br>Autocorrelation<br>Joint Average<br>Cluster Prominence<br>Cluster Shade<br>Cluster Tendency<br>Contrast<br>Correlation<br>Difference Average<br>Difference Entropy<br>Difference Variance<br>Joint Energy<br>Joint Entropy<br>Informational Measure of Correlation (IMC) 1<br>Informational Measure of Correlation (IMC)<br>Inverse Difference Moment (IDM)<br>Maximal Correlation Coefficient (MCC) .<br>Inverse Difference Moment Normalized (IDMN)<br>Inverse Difference (ID) Inverse Difference Normalized (IDN)<br>Inverse Variance<br>Maximum<br>Sum Average<br>Sum Entropy<br>Sum of Squares |
| **Gray Level Size Zone Matrix (GLSZM) Features** | Small Area Emphasis (SAE)<br>Large Area Emphasis (LAE)<br>Gray Level Non-Uniformity (GLN)<br>Gray Level Non-Uniformity Normalized (GLNN)<br>Size-Zone Non-Uniformity (SZN)<br>Size-Zone Non-Uniformity Normalized (SZNN)<br>Zone Percentage (ZP)<br>Gray Level Variance (GLV)<br>Zone Variance (ZV)<br>10. Zone Entropy (ZE)<br>Low Gray Level Zone Emphasis (LGLZE)<br>High Gray Level Zone Emphasis (HGLZE)<br>Small Area Low Gray Level Emphasis (SALGLE)<br>Small Area High Gray Level Emphasis (SAHGLE)<br>Large Area Low Gray Level Emphasis (LALGLE)<br>Large Area High Gray Level Emphasis (LAHGLE) |
| **Gray Level Run Length Matrix (GLRLM) Features**<br>(regional heterogenity) | Short Run Emphasis (SRE)<br>Long Run Emphasis (LRE)<br>Gray Level Non-Uniformity (GLN)<br>Gray Level Non-Uniformity Normalized (GLNN)<br>Run Length Non-Uniformity (RLN)<br>Run Length Non-Uniformity Normalized (RLNN)<br>Run Percentage (RP)<br>Gray Level Variance (GLV)<br>Run Variance (RV)<br>Run Entropy (RE) .<br>Low Gray Level Run Emphasis (LGLRE)<br>High Gray Level Run Emphasis (HGLRE)<br>Short Run Low Gray Level Emphasis (SRLGLE)<br>Short Run High Gray Level Emphasis (SRHGLE)<br>Long Run Low Gray Level Emphasis (LRLGLE)<br>Long Run High Gray Level Emphasis (LRHGLE) |

| | |
|---|---|
| **Neighbouring Gray Tone Difference Matrix (NGTDM) Features** | Coarseness<br>Contrast<br>Busyness<br>Complexity<br>Strength : |
| **Gray Level Dependence Matrix (GLDM) Features** | Small Dependence Emphasis (SDE)<br>Large Dependence Emphasis (LDE)<br>Gray Level Non-Uniformity (GLN)<br>Dependence Non-Uniformity (DN)<br>Dependence Non-Uniformity Normalized (DNUN)<br>Gray Level Variance (GLV) Measures the variance in grey level in the image.<br>Dependence Variance (DV) Measures the variance in dependence size in the image.<br>Dependence Entropy (DE)<br>Low Gray Level Emphasis (LGLE)<br>High Gray Level Emphasis (HGLE<br>Small Dependence Low Gray Level Emphasis (SDLGLE<br>Small Dependence High Gray Level Emphasis (SDHGLE)<br>Large Dependence Low Gray Level Emphasis (LDLGLE)<br>Large Dependence High Gray Level Emphasis (LDHGLE) |

**Table S3.** Results of descriptive analysis for OS, PFS and DCB.

| Variable | OS (p value) | PFS (p value) | DCB (p value) |
|---|---|---|---|
| Age | 0.26 | 0.12 | 0.53 |
| PS (0 vs ≥ 1) | **0.003** | **0.0005** | **0.00009** |
| Tabacco history | 0.29 | 0.98 | 0.48 |
| Histology | 0.72 | 0.72 | 0.58 |
| PD-L1 ≥ 50% | **0.00003** | 0.8 | 0.88 |
| Stage | 0.5 | 0.17 | **0.03** |
| Brain metastasis | 0.2 | 0.83 | 0.7 |

PS : Eastern Cooperative Oncology Group performance status. OS: overall survival; PFS: progression free survival.

**Table S4.** Results of univariate analysis with bootstrap for durable clinical benefit (DCB) (mean AUC, its standard deviation and confidence intervals).

| Feature type | Best feature | DCB |
|---|---|---|
| Clinical | Age | 0.58 0.11 [0.42-0.75] |
| Clinical | PS | 0.70 ± 0.11 [0.429 - 0.818] |
| PET0 Radiomic | DNUN | 0.774 ± 0.09 [0.640 - 0.912] |
| PET1 Radiomic | IMC2 | 0.872 ± 0.05 [0.8 – 0.945] |
| Delta-PET | Total Energy | 0.807 ± 0.10 [0.638 – 0.959] |
| CT0 Radiomic | SALGLE | 0.751 ± 0.10 [0.582 - 0.861] |
| CT1 Radiomic | Busyness | 0.897 ± 0.09 [0.730 – 0.993] |
| Delta-CT | Difference Average | 0.895 ± 0.06 [0.795 - 1] |

PS : Eastern Cooperative Oncology Group performance status; DNUN : Dependence Non-Uniformity Normalized; IMC: Informational Measure of Correlation; SALGLE: Small Area Low Gray Level Emphasis.

A Age

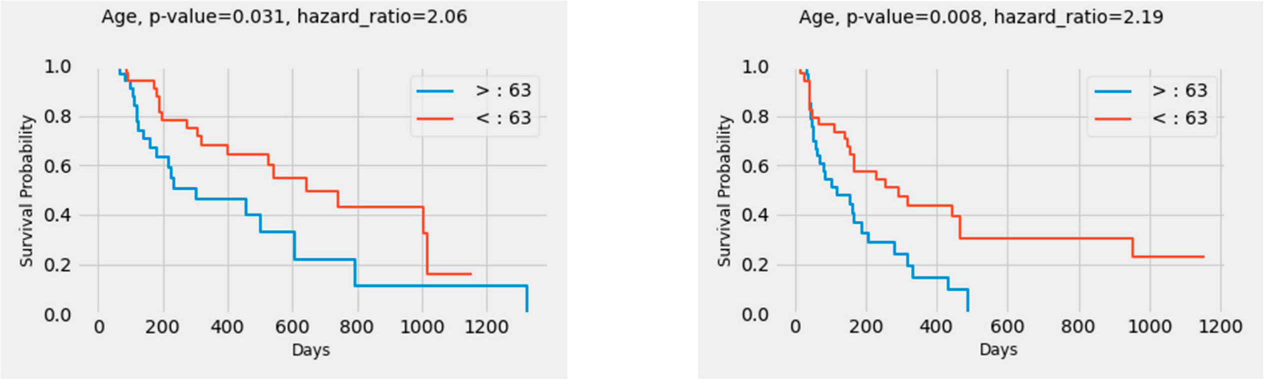


B : Performance status

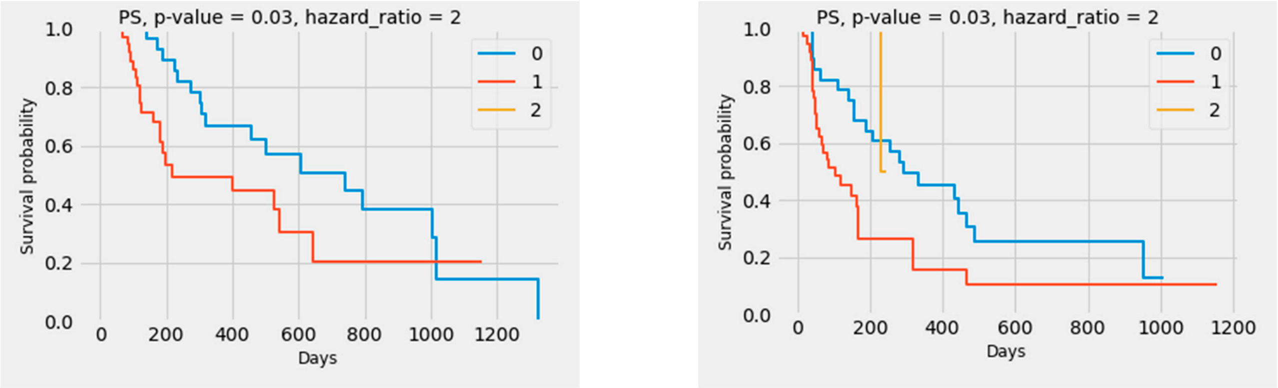


C Histology

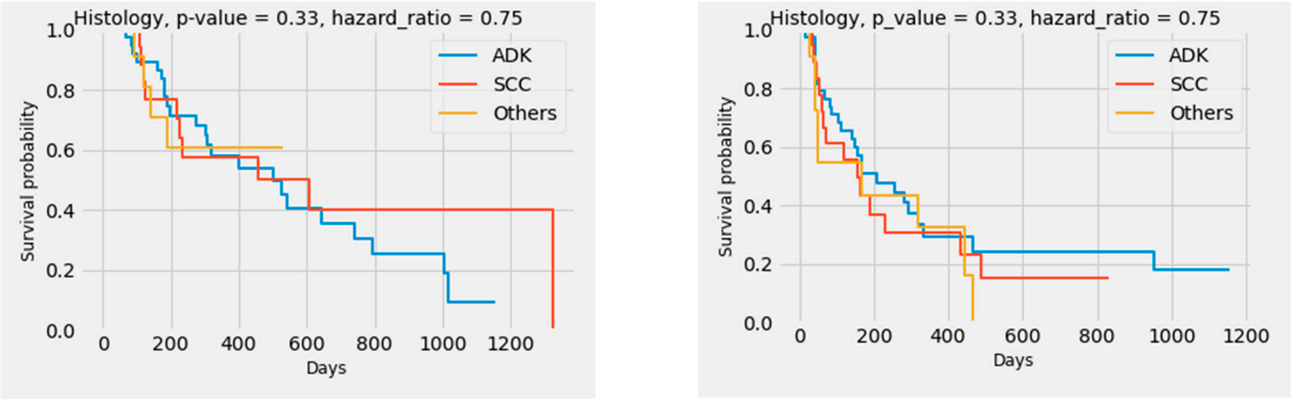


D Smoking history

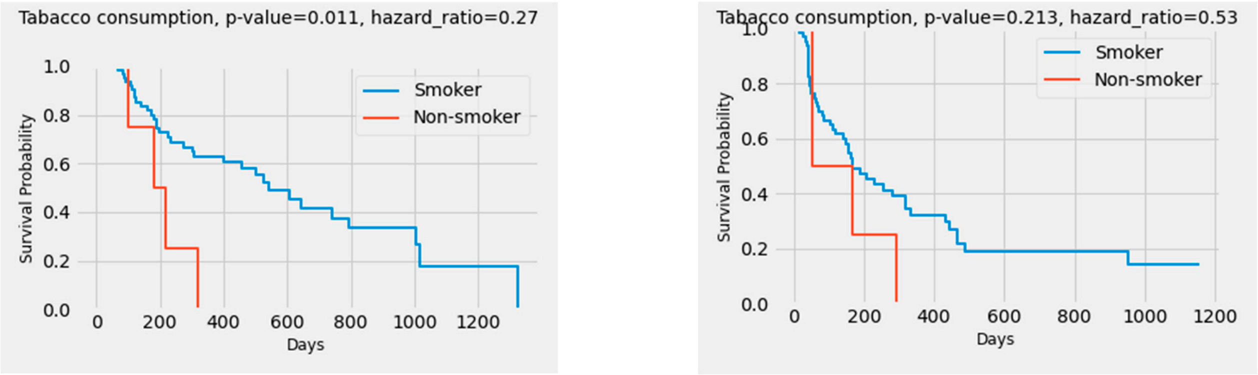


E : Metabolic Tumor volume

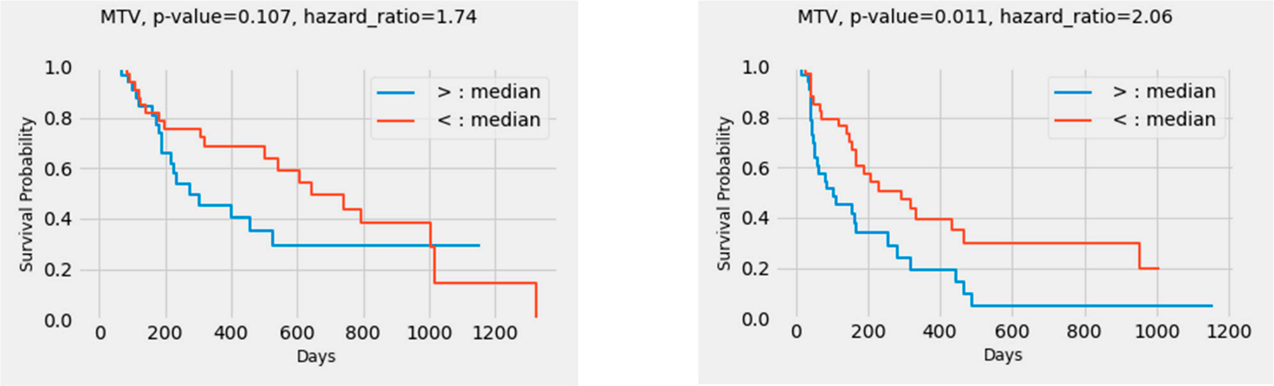


F Total lesion glycolysis

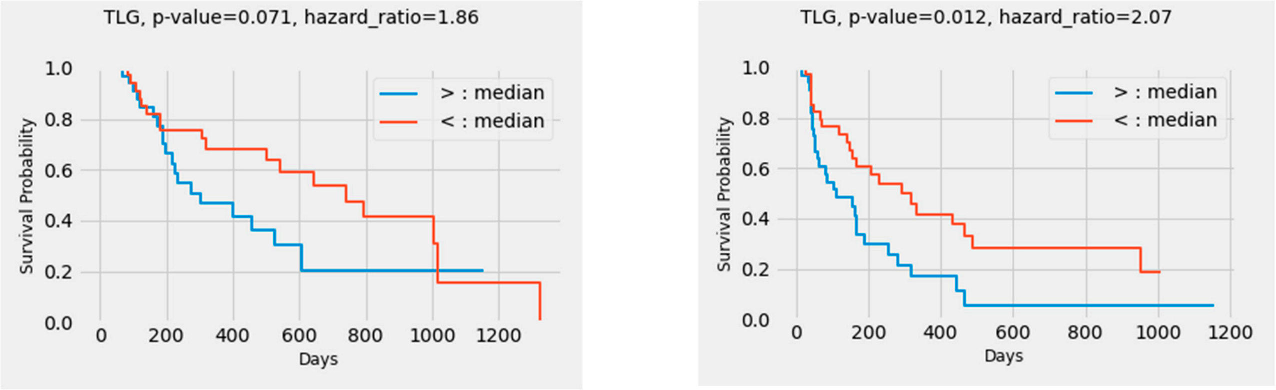


**Figure S1.** Overall Survival (left) and Progression Free Suvival (right) related to clinical and baseline metabolic PET metrics (Kaplan Meier and log rank test).

A – Baseline

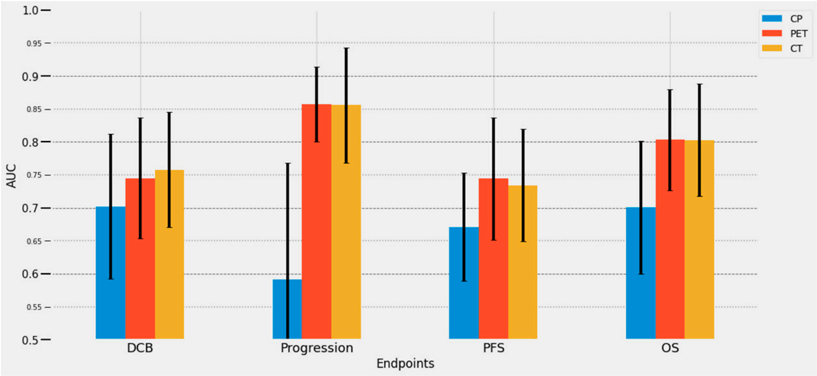


B – 2 months after treatment initiation (PET/CT1)

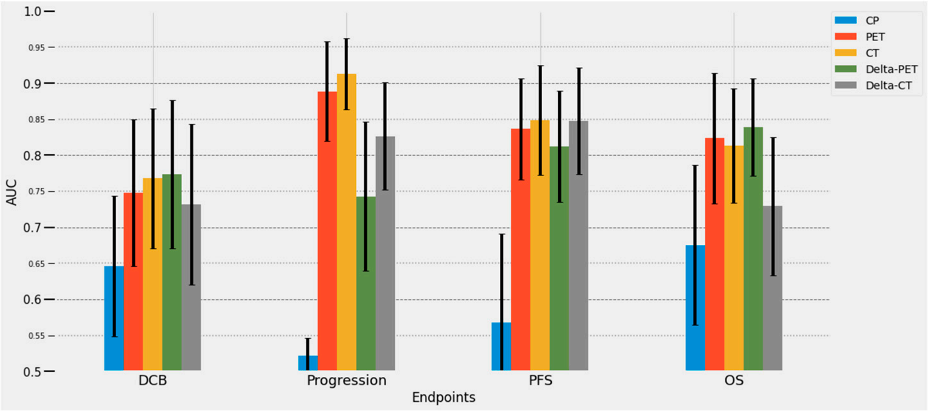


C – 3 months after treatment initiation (PET/CT2)

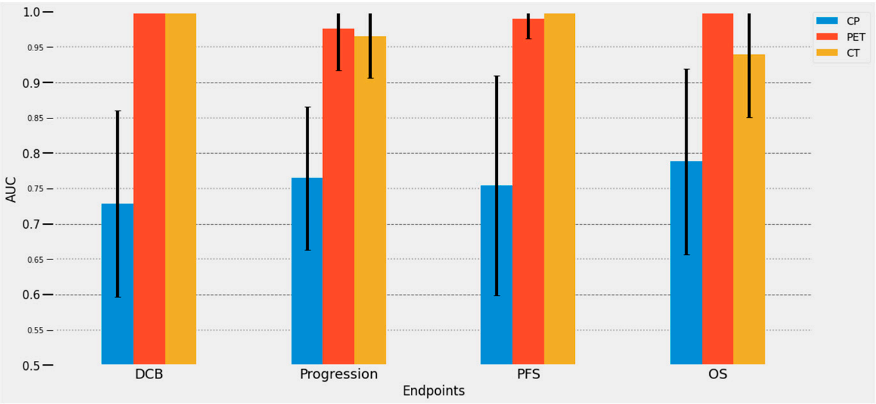


**Figure S2: Prediction of outcome: Best clinical PET and CT parameters performance at baseline and during treatment: best AUC value obtained for the considered parameters using univariate analysis with bootstrap.** *DCB: Durable clinical benefit. OS: overall survival; PFS: progression free survival. OS: overall survival.*

**Table S5.** Kaplan Meier and Cox analysis scores for clinical parameters : (A) Progression-Free Survival, (B) Overall survival.

*A*

| Parameter | Hazard Ratio | CI (95%) | p |
|---|---|---|---|
| **Sex** | 1.36 | 0.7-2.64 | 0.36 |
| **Brain metastasis** | 0.78 | 0.4-1.5 | 0.45 |
| **PS (0 vs 1-2)** | 1.96 | 1.09-3.53 | **0.03** |
| **Tabacco history** | 0.53 | 0.19-1.49 | 0.22 |
| **Stage** | 0.6 | 0.25-1.42 | 0.24 |
| **Histology ADK vs SCC+others** | 0.75 | 0.43-1.33 | 0.33 |
| **Age (cut off : median)** | 2.19 | 1.21-3.96 | **0.01** |

*B*

| Parameter | Hazard ratio | CI (95%) | p |
|---|---|---|---|
| **Sex** | 1.01 | 0.49-2.1 | 0.97 |
| **Metastasis** | 0.8 | 0.37-1.7 | 0.56 |
| **PS (0 vs 1-2)** | 1.73 | 0.89-3.39 | 0.11 |
| **Tabacco** | 0.27 | 0.09-0.79 | **0.02** |
| **Stage** | 0.5 | 0.18-1.43 | 0.2 |
| **Histology ADK vs SCC+others** | 1.19 | 0.6-2.36 | 0.62 |
| **Age** | 2.06 | 1.05-4.01 | **0.03** |

*PS : ECOG performance status; ADK : adenocarcinoma; SCC : squamous cell cancer*

**Table S6: Progression-Free Survival Kaplan Meier and Cox analysis scores for PET radiomics.**

| Radiomic parameter | Hazard Ratio | CI (95%) | p |
|---|---|---|---|
| 0.5_wavelet-HLL_gldm_LargeDependenceHighGrayLevelEmphasis | 2.73 | 1.51-4.93 | 0.001 |
| 64_wavelet-HLH_glcm_ClusterShade | 0.36 | 0.19-0.37 | 0.001 |
| 64_wavelet-HHL_glrlm_LongRunLowGrayLevelEmphasis | 0.37 | 0.2-0.68 | 0.001 |
| 64_wavelet-LLH_firstorder_Skewness | 0.38 | 0.21-0.7 | 0.002 |
| 64_wavelet-LLH_glcm_ClusterShade | 0.39 | 0.22-0.71 | 0.002 |
| 64_log-sigma-3-0-mm-3D_firstorder_Kurtosis | 2.44 | 1.37-4.36 | 0.003 |
| 0.5_wavelet-LLH_glcm_Imc1 | 2.33 | 1.3-4.19 | 0.005 |
| 64_wavelet-HHL_firstorder_Kurtosis | 2.3 | 1.29-4.1 | 0.005 |
| 64_wavelet-LHH_glcm_ClusterProminence | 0.43 | 0.24-0.78 | 0.005 |
| 64_original_glcm_SumSquares | 0.44 | 0.25-0.79 | 0.006 |
| 64_log-sigma-0-5-mm-3D_glcm_ClusterProminence | 0.44 | 0.25-0.79 | 0.006 |
| 64_wavelet-HLL_firstorder_Kurtosis | 2.24 | 1.26-4 | 0.006 |
| Total lesion Glycolysis | 2.07 | 1.16-3.68 | 0.014 |
| Metabolic tumor volume | 2.06 | 1.16-3.64 | 0.013 |

Abreviations : cf list of radiomics parameters.

**Table S7: Overall Survival Kaplan Meier and Cox analysis scores for PET radiomics.**

| Radiomic parameter | Hazard Ratio | CI (95%) | p |
|---|---|---|---|
| **0.5_wavelet-HLL_glcm_ClusterShade** | 0.28 | 0.13-0.61 | 0.002 |
| **64_wavelet-HLH_glcm_ClusterShade** | 0.3 | 0.14-0.65 | 0.002 |
| **64_wavelet-HLH_firstorder_Mean** | 0.33 | 0.16-0.66 | 0.002 |
| **_64_wavelet-HHL_glrlm_LongRunLowGrayLevelEmphasis** | 0.32 | 0.15-0.38 | 0.003 |
| **0.5_wavelet-HHH_glszm_GrayLevelNonUniformityNormalized** | 3.11 | 1.44-6.69 | 0.004 |
| **64_wavelet-LHH_glcm_ClusterProminence** | 0.33 | 0.15-0.71 | 0.004 |
| **64_wavelet-HLL_firstorder_Kurtosis** | 2.8 | 1.38-5.7 | 0.004 |
| **Total lesion Glycolysis** | 1.86 | 0.94-3.7 | 0.074 |
| **Metabolic tumor volume** | 1.74 | 0.88-3.45 | 0.11 |

Abreviations : cf list of radiomics parameters.

**Table S8: Progression-Free Survival Kaplan Meier and Cox analysis scores for CT radiomics.**

| Radiomic | Hazard Ratio | CI l(95%) | p |
|---|---|---|---|
| Interpolated1_32_wavelet-HLH_glszm_SizeZoneNonUniformityNormalized | 0.28 | 0.15-0.51 | 3E-05 |
| Original_64_wavelet-LLL_glszm_GrayLevelNonUniformityNormalized | 3.02 | 1.66-5.5 | 3E-05 |
| Original_64_original_glcm_ClusterProminence | 0.36 | 0.2-0.66 | 0.0009 |
| Interpolated1_32_wavelet-HHL_glszm_SizeZoneNonUniformityNormalized | 0.4 | 0.23-0.72 | 0.002 |
| Original_32_wavelet-HLH_glcm_InverseVariance | 2.46 | 1.38-4.4 | 0.002 |
| Original_64_original_glcm_Imc1 | 2.45 | 1.36-4.43 | 0.003 |
| Original_32_log-sigma-3-0-mm-3D_glszm_GrayLevelVariance | 0.41 | 0.23-0.74 | 0.003 |
| Original_64_log-sigma-1-0-mm-3D_glrlm_LongRunLowGrayLevelEmphasis | 0.4 | 0.22-0.74 | 0.003 |
| Original_32_log-sigma-2-0-mm-3D_glszm_LowGrayLevelZoneEmphasis | 0.42 | 0.23 | 0.004 |
| Original_32_wavelet-LLL_glcm_InverseVariance | 2.38 | 1.32 | 0.004 |
| Original_64_log-sigma-3-0-mm-3D_glcm_Imc2 | 0.42 | 0.23 | 0.04 |
| 64_wavelet-LLH_firstorder_Skewness | 0.43 | 0.24 | 0.005 |
| Interpolated1_32_wavelet-LLH_glszm_SizeZoneNonUniformityNormalized | 0.43 | 0.24 | 0.005 |
| Original_64_log-sigma-2-0-mm-3D_firstorder_Skewness | 0.43 | 0.24 | 0.005 |
| Interpolated1_64_wavelet-HHH_firstorder_Median | 2.29 | 1.27 | 0.006 |
| Original_32_wavelet-LLL_glrlm_RunEntropy | 0.44 | 0.24 | 0.006 |
| Original_64_log-sigma-2-0-mm-3D_glcm_ClusterShade | 0.45 | 0.25 | 0.006 |

Abreviations : cf list of radiomics parameters.

**Table S9: Overall Survival Kaplan Meier and Cox analysis scores for CT radiomics.**

| Radiomic | Hazard Ratio | CI (95%) | p |
|---|---|---|---|
| HLH_glszm_SizeZoneNonUniformityNormalizedInterpolated1_32_wavelet | 0.34 | 0.16-.68 | 0.003 |
| 32_wavelet-HLH_glcm_InverseVariance | 2.71 | 1.36-5.38 | 0.005 |
| Interpolated1_32_wavelet-LLH_glszm_SizeZoneNonUniformityNormalized | 0.37 | 0.18-0.75 | 0.006 |
| Original_64_wavelet-HHL_glszm_LargeAreaEmphasis | 2.79 | 1.33-5.86 | 0.007 |

Abreviations : cf list of radiomics parameters.

**Table S10: Radiomics Quality Score**

| Item | Points | Current Study |
|---|---|---|
| Image protocol quality - well-documented image protocols (for example. contrast. slice thickness. energy. etc.) and/or usage of public image protocols allow reproducibility/replicability | ☑ protocols well documented (+1)<br>☑ public protocol used (+1)<br>☐ none | 2 |
| Multiple segmentations - possible actions are: segmentation by different physicians/algorithms/software. perturbing segmentations by (random) noise. segmentation at different breathing cycles. Analyse feature robustness to segmentation variabilities | ○ yes (+1)<br>○ no | 0 |
| Phantom study on all scanners - detect inter-scanner differences and vendor-dependent features. Analyse feature robustness to these sources of variability | ○ yes (+1)<br>◉ no | 0 |
| Imaging at multiple time points - collect images of individuals at additional time points. Analyse feature robustness to temporal variabilities (for example. organ movement. organ expansion/shrinkage) | ○ yes (+1)<br>◉ no | 0 |
| Feature reduction or adjustment for multiple testing - decreases the risk of overfitting. Overfitting is inevitable if the number of features exceeds the number of samples. Consider feature robustness when selecting features | ◉ Either measure implemented (+3)<br>○ Neither measure implemented (-3) | 3 |
| Multivariable analysis with non radiomics features (for example. EGFR mutation) - is expected to provide a more holistic model. Permits correlating/inferencing between radiomics and non radiomics features | ◉ yes(+1)<br>○ no | 1 |
| Detect and discuss biological correlates - demonstration of phenotypic differences (possibly associated with underlying gene–protein expression patterns) deepens understanding of radiomics and biology | ○ yes (+1)<br>◉ no | 0 |

| | | |
|---|---|---|
| Cut-off analyses - determine risk groups by either the median. a previously published cut-off or report a continuous risk variable. Reduces the risk of reporting overly optimistic results | ◉ yes (+1)<br>○ no | 1 |
| Discrimination statistics - report discrimination statistics (for example. C-statistic. ROC curve. AUC) and their statistical significance (for example. p-values. confidence intervals). One can also apply resampling method (for example. bootstrapping. cross-validation) | ☑ a discrimination statistic and its statistical significance are reported (+1)<br>☑ a resampling method technique is also applied<br>☐ none | 2 |
| Calibration statistics - report calibration statistics (for example. Calibration-in-the-large/slope. calibration plots) and their statistical significance (for example. P-values. confidence intervals). One can also apply resampling method (for example. bootstrapping. cross-validation) | ☑ a calibration statistic and its statistical significance are reported (+1)<br>☑ a resampling method technique is applied<br>☐ none | 2 |
| Prospective study registered in a trial database - provides the highest level of evidence supporting the clinical validity and usefulness of the radiomics biomarker | ○ yes (+7)<br>◉ no | 0 |
| Validation - the validation is performed without retraining and without adaptation of the cut-off value. provides crucial information with regard to credible clinical performance | ☐ No validation (-5)<br>☑ validation is based on a dataset from the same institute (+2)<br>☐ validation is based on a dataset from another institute (+3)<br>☐ validation is based on two datasets from two distinct institutes (+4)<br>☐ the study validates a previously published signature (+4)<br>☐ validation is based on three or more datasets from distinct institutes (+5) | 2 |

| | | |
|---|---|---|
| Comparison to 'gold standard' - assess the extent to which the model agrees with/is superior to the current 'gold standard' method (for example. TNM-staging for survival prediction). This comparison shows the added value of radiomics | ☑ yes (+2)<br>☐ no | 2 |
| Potential clinical utility - report on the current and potential application of the model in a clinical setting (for example. decision curve analysis). | ☑ yes (+2)<br>☐ no | 2 |
| Cost-effectiveness analysis - report on the cost-effectiveness of the clinical application (for example. QALYs generated) | ☑ yes (+2)<br>☐ no | 2 |
| Open science and data - make code and data publicly available. Open science facilitates knowledge transfer and reproducibility of the study | ☐ scans are open source (+1)<br>☐ region of interest segmentations are open source (+1)<br>☐ the code is open sourced (+1)<br>☐ Radiomics features are calculated on a set of representative ROIs and the calculated features and representative ROIs are open source (+1) | 0 |
| **Total** | **36** | **19** |
